# Many kinds of oxidized proteins are present more in the urine of the elderly

**DOI:** 10.1186/s12014-022-09360-2

**Published:** 2022-06-22

**Authors:** Yongtao Liu, Xuanzhen Pan, Yijin Bao, Lilong Wei, Youhe Gao

**Affiliations:** 1 Department of Biochemistry and Molecular Biology, Beijing Key Laboratory of Gene Engineering Drug and Biotechnology, Beijing Normal University, Beijing, 100875 China; 2 Clinical Laboratory, China-Japan Friendship Hospital, Beijing, 100029 China

**Keywords:** Aging, Modifications, Oxidations, Urine, pFind, Mass spectrometry

## Abstract

**Background:**

Many studies have shown an association between aging and oxidation. To our knowledge, there have been no studies exploring aging-related urine proteome modifications. The purpose of this study was to explore differences in global chemical modifications of urinary protein at different ages.

**Methods:**

Discovery (n=38) cohort MS data including children, young and old groups were downloaded from three published studies, and this data was analyzed using open-pFind for identifying modifications. Verification cohort human samples (n=28) including young, middle-aged, and old groups, rat samples (n=7) at three-time points after birth, adulthood, and old age were collected and processed in the laboratory simultaneously based on label-free quantification combined with pFind.

**Results:**

Discovery cohort: there were 28 kinds of differential oxidations in the old group that were higher than those in the young or children group in. Verification cohort: there were 17 kinds of differential oxidations of 49 oxidized proteins in the middle and old groups, which were significantly higher than those in the young group. Both oxidations and oxidized proteins distinguished different age groups well. There were also 15 kinds of differential oxidations in old age higher than others in the rat cohort. The results showed that the validation experiment was basically consistent with the results of the discovery experiment, showing that the level of oxidized proteins in urine increased significantly with age.

**Conclusions:**

Our study is the first to show that oxidative proteins occur in urine and that oxidations are higher in older than younger ages. Perhaps improving the degree of excretion of oxidative protein in vivo through the kidney is helpful for maintaining the homeostasis of the body’s internal environment, delaying aging and the occurrence of senile diseases.

**Supplementary Information:**

The online version contains supplementary material available at 10.1186/s12014-022-09360-2.

## Background

Aging is a process that everyone must face. For almost a century on, scientists have been constantly exploring the mysteries of aging and trying to find ways to slow down aging. Some studies have found that oxidative stress exacerbates aging and age-related diseases [1–6], limiting healthful longevity. Therefore, protection against oxidative stress is a common mechanism mediating the phenotype observed in animal models of longevity [5].

Protein oxidation product is one of the oxidative stress indicators. In this study, a series of oxidations and oxidative proteins were found to change with aging from urinary proteome in human and rat cohorts, which may provide a distinctive perspective to predict the evolving trends in the aging of human, even reversing the aging progress. Most age-related pathologies and aging processes are accompanied by a dysregulation of ubiquitin-proteasomal system (UPS), leading to the accumulation of some damaged proteins [7]. These damaged proteins with structural changes could be excreted by the body. Urine filtered plasma proteins originate from distal organs, including the brain, etc, not only the kidney [8–10]. Recent studies indicate that urine offers another promising clinically viable matrix for many diseases as it can be frequently and noninvasively collected in large volumes, especially providing diagnostic and prognostic opportunities [10–14]. Nevertheless, only a handful of PTMs have been studied and applied in urinary biomarker researches [15, 16]. Here, for the first time, we explore the relationship between urinary protein modifications and aging. We applied a mass spectrometry-based, data-dependent acquisition (DDA) with a sequence-tag-based search engine (Open-pFind) [17, 18] to discover modifications, through limit-search [19, 20] as refined search analyses and random grouping calculations to achieve technical verification. Then, we also validated the results in another human cohort and rat cohort as biological duplications.

## Methods

### Acquisition of experimental samples and data

The discovery cohort involved urine samples from 38 healthy individuals, including children, young and senior groups. Children (n=22) and senior (n=6) RAW data were download from published studies [21, 22], and data from the young group (n=10) were obtained from existing data in our laboratory. Validation cohorts were combined with human urine samples (n=28) and male rat samples (n=21). Human cohort included young (n=8), middle-aged (n=8) and old (n=12) groups. All 29 healthy individuals conformed to periodic physical examination with passing medical tests, and their detailed parameters and information are available in Additional file 1: Table S1. These samples were collected from the clinical laboratory of China-Japan Friendship Hospital during a fasting physical examination. This study’s ethics approval was approved by the China-Japan Friendship Hospital review boards, and each participant signed informed consent. These urine samples were collected and stored in the same environment. We tried to avoid samples being exposed to air and reduced the time the sample stayed at room temperature. Finally, samples were frozen in a −80 ℃ fridge refrigerator, and then processed together.

The rat cohort includeed seven male rats of which the mother and father were born from the same brood of the same parents. We collected data from their three developmental periods including childhood (27 days), entering adulthood (240 days), and reproductive senescence (600 days) [23–25]. All rats were bred from birth to the indicated day, with the same fodders, and they lived in the same environment. The animal experiments were approved by the Ethics Review Committee of the Institute of College of Life Science, Beijing Normal University, China. Male rats’ parents were purchased from Beijing Charles River Laboratory. The rats were acclimated to the environment for one week before the experiment. All experimental animals were utilized following the “Guidelines for the Care and Use of Laboratory Animals” issued by the Beijing Office of Laboratory Animal Management (Animal Welfare Assurance Number: ACUC-A02-2015-004). These urine samples were collected in the same environment, frozen in −80℃ fridge refrigerators, and then processed together.

### Sample preparation for label-free analysis

The urine samples were reacted with 20 mmol/L dithiothreitol (DTT) at 37 ℃ for 1 h to denature the disulfide bonds in the protein structure, followed by the addition of 55 mmol/L iodoacetamide (IAA) in the dark for 30 min to alkylate the disulfide bond site. Precipitated the supernatant with three-fold volumes of pre-cooled acetone at −20 °C for 2–4 h, and then centrifuged at 12,000 ×*g* for 30 min at 4 °C to obtain protein precipitate. The pellet was then resuspended in an appropriate amount of protein solubilization solution (8 mol/L urea, 2 mol/L thiourea, 25 mmol/L DTT, and 50 mmol/L Tris). The protein-concentrated solution was measured using Bradford analysis. By using the filter-assisted sample preparation (FASP) method, 100 µg of each sample was digested with trypsin (Trypsin Gold, Mass Spec Grade, Promega, Fitchburg, WI, USA) at a ratio of 50:1. After digestion with trypsin at 37°C for 14 h, 10% formic acid solution was added to the solution to terminate the enzymolysis, and the peptide solution was obtained after centrifugation through a 10 kDa ultrafiltration tube. The concentration of the peptide was determined using the BCA method and passed through a vacuum centrifugal concentrator (Thermo Fisher, USA), and the dried peptides were sealed and stored at −80 °C. Additional file 1: Table S7 shows the urine sample processing methods of the published studies in the literature, and the comparison with this method.

### Liquid chromatography and mass spectrometry

Before analysis of urine samples of healthy young individuals, the dried peptide samples should be dissolved in 0.1% FA (formic acid) for liquid chromatography-mass spectrometry analysis, the final concentration should be controlled at 0.1 μg/μL, and each sample should be analyzed with 1 μg peptide. For DDA experiments, iRT (indexed retention time; Biogenesis, Switzerland) calibration peptides were spiked into the sample. Thermo EASY-nLC1200 chromatography system was loaded to Pre-column and the analytical column. Proteome data was collected by the Thermo Orbitrap Fusion Lumos mass spectrometry system (Thermo Fisher Scientific, Bremen, Germany). Liquid chromatography analysis method: pre-column: 75 μm×2 cm, nanoViper C18, 2 μm, 100 Å; analytical column: 50 μm×15 cm, nanoViper C18, 2 μm, 100 Å; injection volume: 10 μL, flow rate: 250 nL/min. The mobile phase configuration is as follows, phase A: 100% mass spectrometric grade water (Fisher Scientific, Spain)/1% formic acid (Fisher Scientific), phase B: 80% acetonitrile (Fisher Scientific, USA)/20% water/1‰ formic acid, 120 min gradient elution: 0 min, 3% phase B; 0–3 min, 8% phase B; 3–93 min, 22% phase B; 93–113 min, 35% phase B; 113–120 min, 90% phase B; mass spectrometry method, ion source: nanoESI, spray voltage: 2.0 kV, capillary temperature: 320 ℃, S-lens RF Level: 30, resolution setting: level 1 (Orbitrap) 120,000 @m/z 200, Level 2 30,000 (Orbitrap) @m/z 200, precursor ion scan range: m/z 350-1350; product ion scan range: from m/z 110, MS1 AGC: 4e5, charge range: 2–7, Ion implantation time: 50 ms, MS2 AGC: 1e5, ion implantation time: 50 ms, ion screening window: 2.0 m/z, fragmentation mode: high energy collision dissociation (HCD), energy: NCE 32, Data-dependent MS/MS : Top 20, dynamic exclusion time: 15s, internal calibration mass: 445.12003. Additional file 1: Table S8 shows the urine sample data collection methods of published studies in the literature and compares them with our method.

### Analysis of urinary proteomes with the MaxQuant and Perseus software tool

The validation cohorts included 28 healthy individuals and 7 rats, allowing for robust statistics when performing label-free quantitative comparisons. Each sample was run in technical triplicates for more reliable generation of three RAW files that contained all acquired full MS and MS2 spectra. Base peak chromatograms were inspected visually in Xcalibur Qual Brower version 4.0.27.19 (Thermo Fisher Scientific). RAW files were processed by MaxQuant version 1.6.17.0 (http://www.maxquant.org) using default parameters unless otherwise specified [21, 26–29]. All RAW files of one species were analyzed together in a single MaxQuant run. Database searches were performed using the Andromeda search engine included with the MaxQuant release [30] with the Uniprot human and rat sequence database (November 27, 2020; 196,211 sequences; April 17, 2021; 36,181 sequences). Precursor mass tolerance was set to 4.5 ppm in the main search, and fragment mass tolerance was set to 20 ppm. Digestion enzyme specificity was set to Trypsin/P with a maximum of two missed cleavages. A minimum peptide length of seven residues was required for identification. Up to five modifications per peptide were allowed; acetylation (protein N-terminal) and oxidation (Met) were set as variable modifications, and carbamidomethyl (Cys) was set as fixed modification. No Andromeda score threshold was set for unmodified peptides. A minimum Andromeda score of 40 was required for modified peptides. Peptide and protein false discovery rates (FDR) were both set to 1% based on a target-decoy reverse database. Proteins that shared all identified peptides were combined into a single protein group. If all identified peptides from one protein were a subset of identified peptides from another protein, these proteins were combined into that group. Peptides that matched multiple protein groups (“razor” peptides) were assigned to the protein group with the most unique peptides. “Match between run” based on accurate m/z and retention time was enabled with a 0.7 min match time window and 20 min alignment time window. Label-free quantitation (LFQ) was performed using the MaxLFQ algorithm built into MaxQuant [31]. Peaks were detected in Full MS, and a three-dimensional peak was constructed as a function of peak centroid m/z (7.5 ppm threshold) and peak area over time. Following de-isotoping, peptide intensities were determined by extracted ion chromatograms based on the peak area at the retention time with the maximum peak height. And peptide intensities were normalized to minimize overall proteome differences based on the assumption that most peptides do not change in intensity between samples. Protein LFQ intensity was calculated from the median of pairwise intensity ratios of peptides identified in two or more samples and adjusted to the cumulative intensity across samples. Quantification was performed using razor and unique peptides, including those modified by acetylation (protein N-terminal) and oxidation (Met). A minimum peptide ratio of one was required for protein intensity normalization, and “Fast LFQ” was enabled. Only proteins that were quantified by at least two unique peptides were used for analysis. RAW data of mass spectrometry are available in iProX Datasets under the Project ID: IPX0002313003 (https://www.iprox.org/page/HMV006.html).

Data processing was performed Perseus version 1.6.14.0 (http://www.perseus-framework.org) [32, 33]. Contaminants, reverse, and protein groups identified by a single peptide were filtered from the dataset. FDR was calculated as the percentage of reverse database matches out of total forward and reverse matches. Protein group LFQ intensities were log_2_ transformed to reduce the effect of outliers. Protein groups missing LFQ values were assigned values using imputation. Missing values were assumed to be biased towards low abundance proteins that were below the MS detection limit, referred to as “missing not at random”, an assumption that is frequently made in proteomics studies [27, 33, 34]. Imputation was performed separately for each group from a distribution with a width of 0.3 and a downshift of 1.8.

### Open search to uncover and identify global modifications and refined search for verification in pFind software tool

RAW data files were searched against* Homo sapiens *Uniprot canonical database. Database searches were performed with pFind studio (Version 3.1.5) [17], using default parameters unless otherwise specified [35–37]. Precursor ion mass and fragmentation tolerance were set as 10 ppm and 20 ppm, respectively. The maximum number of modifications allowed per peptide was three, as was the maximum number of missed cleavages allowed. The minimum peptide length was set to six amino acids. To discover global modifications, the Open Search was selected (Additional file 1: Fig. S6 shows Open-search and refined-search detailed parameters). For protein-level analysis, mass shifts of +15.9949 Da (methionine oxidation) and +28.0313 Da (dimethylation, Light, N-term/K) were searched as variable modifications; mass shifts of +57.0214 Da (Carbamidomethyl cysteine) was searched as fixed modifications. The FDRs were estimated by the program from the number and quality of peptide-spectrum-match (PSM) to the decoy database. The FDRs at spectrum, peptide, and protein levels were < 1%, and the Q-value at the protein level was less than 1%. Data are analyzed using both forward and reverse database retrieval strategies.

Refined-search did not select Open Search option. And for fixed modifications, mass shifts of +28.0313 Da (dimethylation, Light, N-term/K), +57.0214 Da (Carbamidomethyl cysteine), +15.9949 Da (methionine oxidation), −17.0265 Da (Pyro-glu from Q) and +114.0429 Da (cystine glycineglycine/double carbamidomethylation) were searched, which are top five in modifications proportion rank of open-search results. Meanwhile, selecting them as fixed modifications in the next refined-search can reduce the false-positive rate of validation results as a quantity control. All oxidations were searched as variable modifications, including mass shifts of +15.9949 Da (oxidations), +31.9898Da (Dioxidations), and +47.9847 Da (Trioxidations).

Quantification of heavy to light ratios (R_H/L_) was performed using pQuant as previously described [36, 38], which directly uses the RAW files as the input. pQuant calculates R_H/L_ values based on each identified MS scan with a 15 ppm-level m/z tolerance window and assigns an interference score (Int. Score, also known as confidence score) to each value from zero to one. In principle, the lower the calculated Int. Score, the less co-elution interference signal was observed in the extracted ion chromatograms. In this regard, the median values of oxidatively modified peptide ratios with σ less than or equal to 0.5 were considered to calculate site-level ratios. For each independent experiment, only proteins identified by two or more distinct peptides with quantified PSM R_H/L_ values were retained for further analysis. In this regard, the R_H/L_ value of each identified protein was calculated as the median of all corresponding PSM R_H/L_ values. For site-level analysis, a differential modification of 15.9949 Da on probe-derived modification was used for quantifying PSM R_H/L_ values. For each independent experiment, The R_H/L_ value of each oxidatively modified site was calculated as the median of all corresponding PSM R_H/L_ values.

### Statistical analysis

We required the proteins to be removed if the CVs of the protein intensity in QC samples were more than 30 %. Log ratios were calculated as the difference in log_2_ LFQ intensity averages between different age groups. Two-tailed, unpaired, heteroscedastic Student’s t-test calculations were used in statistical tests as histograms of LFQ intensities showed that all datasets approximated normal distributions. *P*-value<0.05 was considered statistically significant. Base 2-fold-change values for ratios <1 are represented as negative reciprocals of the ratios.

For quantify modifications, after open-search and refined-search, we obtain “pd.all_result” document, which obtains the number of identified peptides and the protein groups to which these modifications belong. As well-known, multiple kinds of modifications can occur on a single peptide, and the same kind of modifications can occur on different sites of the same peptide (Additional file 1: Fig. S7a). However, information on the total number of sites where a modification occurs or on how many types of peptides it occurs on is not provided directly. Therefore, we wrote a program to extract this information from the “pd.all_result” document. The program codes are available (Supplementary site calculate method code). Most of the significantly differential modifications in “Species” are basically in line with “Sites” (Additional file 1: Fig. S7b). Comparisons of modifications proportions (one modification’s sites/total modifications sites in this sample) between different groups were conducted using two-sided paired t-tests. The differential modifications were selected according to a *P-value* < 0.05 and a fold change >2 or <0.5.

Meanwhile, we also used a mini-program (designed and developed by our lab) for random grouping testing to eliminate interruptions between groups. Briefly, the program is based on “permutation and combination”. For example, for a bunch of m pieces of data divided into n groups, there are ${C}_{m}^{n}$ kinds of combinations, our program will calculate the differential modifications or proteins probability of occurring in these random combinations, and the amount if they are still differential (if they satisfy the above conditions).

### Bioinformatics analysis

Unsupervised clustering and hierarchical clustering analysis were performed using the ‘Wu Kong’ platform (https://www.omicsolution.org/wkomics/main/) [39]. The distance algorithms of both rows and columns were performed by correlation linkage algorithm. ROC curves and PCA analysis were performed using Hiplot platform (https://hiplot.com.cn/basic/roc). The differential proteins were analyzed by Gene Ontology (GO) based on the biological process, cellular component, molecular function, and KEGG using DAVID (https://david.ncifcrf.gov/) [40]. Canonical pathways and diseases or functions annotation for significantly differentially expressed proteins were generated by g:Profiler toolkit[41] (https://biit.cs.ut.ee/gprofiler/gost), functional profiling of each protein set was conducted using the GO [42], KEGG [43] and WikiPathways databases [44], the full set of GO molecular function, GO biological process, KEGG and WikiPathway terms are provided in Additional Figs. Protein interaction network analysis was performed using STRING (https://string-db.org/cgi/input.pl) based on the STRING database [45]. T-test, analysis of variance, Mann-Whitney U-test, and Kruskal-Wallis test were used to evaluate the statistical comparison between groups. Statistical analysis was performed using GraphPad Prism v9.0. *p*-value <0.05 was considered significant.

Based on the huge amount of calculation required to limit the algorithm search, this article by virtue of the supercomputing service provided by the platform of the Supercomputing Center of Beijing Normal University. The hardware information was as follows: Windows Computing Platform (8480-4 Windows VM1), configure 4 Xeon Platinum 8160 processors, each processor has 24 cores, each CPU main frequency is 2.1GHz, memory speed is 2666 MHz, and each node is configured with 1TB DDR4 2666 ECC REG memory (32*32GB). Total hard disk capacity ≥ 9 TB, virtual machine configuration: Windows 10, number of cores: 48, memory: 512 GB, total hard disk capacity ≥ 2 TB (expandable).

## Results

### Cohorts characteristics

In discovery cohort, the healthy people samples consisted of children, the young and senior groups (n=38). The children and senior groups were from published data [21, 22], the young group came from existing data in our laboratory. The validation collection (n=28) contained young and middle-aged and the old individuals to eliminate environmental and technological differences. Finally, we collected seven male rats’ urine samples when they were in childhood, entering adulthood, and undergoing reproductive senescence [23–25]. The seven rats’ mothers and the fathers were born from the same brood of the same parents. Detailed information of healthy cohorts is presented in Additional file 1: Table S1.

### Overview of discovery cohort modifications in urinary proteomics

**Fig. 1:**
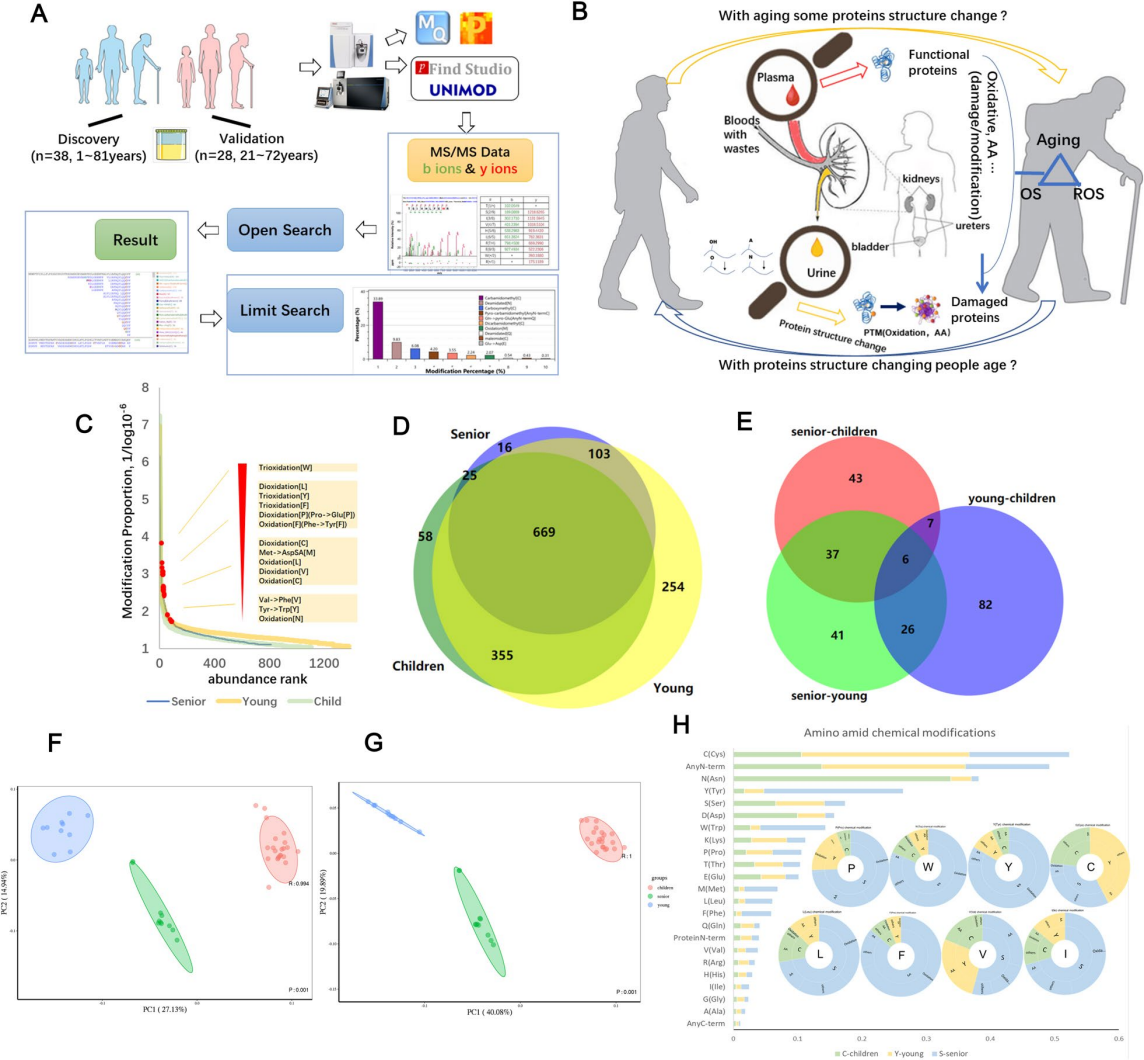
Overview of discovery cohort for chemical modifications in urinary proteomics. **A** Experimental flow chart, schematic representation of analysis of the urinary proteome. Discovery cohorts contained 38 samples from 1 to 81 years, and validation cohorts contained 29 samples from 21 to 72 years. First, we use D open-search to find out modifications of aging signature, next we used refined-search to retrieve these modifications for verification. **B** Conjectures about the relationship between aging and post-translational modification of proteins. Changes in the structure of some proteins are not only the cause of aging but also the phenomenon accompanying aging, and these changes in the structure of proteins damage their functions and are even harmful to the body, they are excreted in the urine. **C** Median modification abundance distribution as calculated from MS intensities of quantified sites of each modification. **D** Intersections between waves of aging global modifications in discovery cohorts. **E** Venn diagram of significantly changed modifications. The overlap between waves of aging modifications (n=242; *p*-value<0.05, fold change>2 or <0.5), significance was tested using the t-test. **F** and** G**, PCA of total modifications (R:0.994, P:0.001) and PCA of significantly changed modifications (R:1, P:0.001). **H** Statistics on the number and proportion of various amino acid site modifications (In order to better display the data, Carbamidomethyl [C] and Oxidation [M] were removed because they accounted for almost 60% of the total number of artefacts.) between the three age groups. The abscissa is the ratio of the number of various amino acid modification sites to the sum of the total modifications’ sites identified. The pie chart shows the proportion of the same amino acid modification between different age groups, the detailed content of each part has been placed in the schedule

Relying on the label-free quantitative proteome method, the experimental results of urine samples were obtained by LC-MS/MS analysis. After retrieving data (RAW) based on pFind software, the analysis results were shown in pBuild. The results were sorted and counted (flowchart in Fig. 1a). Here, we found that some oxidations and amino acid (AA) substitutions were higher in the old group than others. These proteins’ structures change with aging and in turn, their structural variation also influences aging (Fig. 1b). First, we used Open-search method to identify a total of 1480 modifications in 38 samples; these modifications occurred at 20 amino acids and the N and C terminals in a protein chain. Additional file 1: Table S2 showed the information on the proportion of modifications occurring at each amino acid. The quantified modification intensities spanned over seven orders of magnitude, in which the top ten most abundant proteins contributed 81–91% of the total modification intensity of the entire (Fig. 1c). The identified modification numbers among three different ages were shown in Fig. 1d. The overlap between modifications changing at children, young and senior stages was significant using a two-tailed test (*p*-value < 0.05, fold change >2 or <0.5), and all significantly differential modifications were 242 (Fig. 1e). Normed principal-component analysis was used to characterize changes of the aging signature modifications by total modifications (Fig. 1f) and significantly differential modifications (Fig. 1g) in sites, respectively. Both displayed effective separation of different age groups. As shown in the histogram of Fig. 1h, we calculated the ratio of different amino acid modifications according to the ratio (modification sites number/ peptide number) of each modification in each age group. At the same time, we found that among the non-artificial modifications of proline(Pro, P), tryptophan(Trp, W), tyrosine(Tyr, Y), cysteine(Cys, C), leucine(Leu, L), phenylalanine(Phe, F), valine(Val, V) and isoleucine(Ile, I), oxidations and amino acid substitutions were dominant, especially in proline, tryptophan, tyrosine, leucine, phenylalanine and isoleucine, the oxidations rate of the senior group was higher than that in other age groups, as shown in the pie chart (Fig. 1h).

### Oxidations and AA substitutions changing in the discovery cohort

**Fig. 2:**
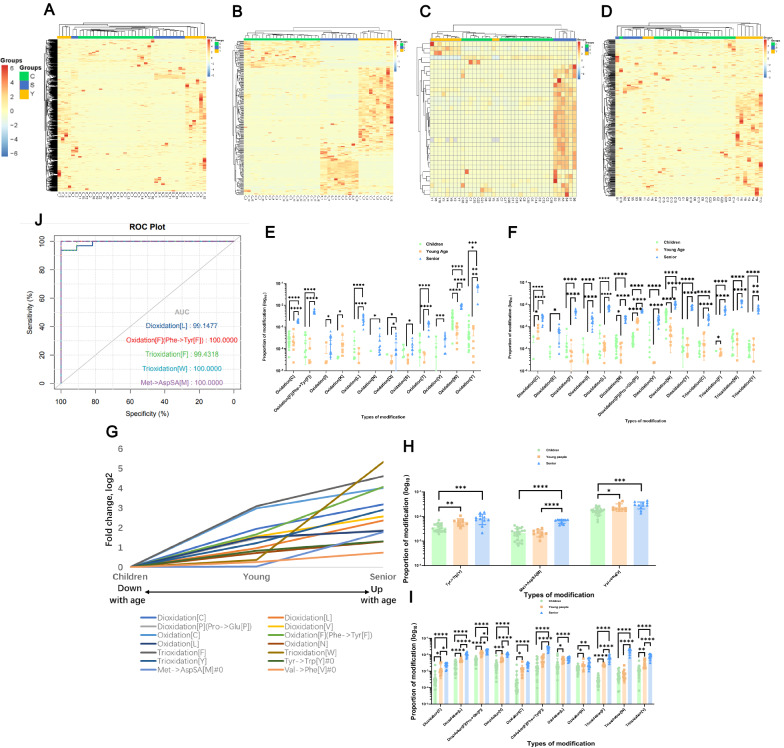
Oxidations and AA substitutions changing with aging in discovery cohort. **A** Unsupervised cluster analysis results of the total modifications for three groups samples. Children group covers with green, and young age group covers with yellow, the senior group covers with blue. **B** Heatmap depicting the levels of differentially identified modifications in discover cohorts with different age groups. The graphs show the relative intensity of differentially expressed modifications. Proteins included in the heatmap meet the requirement that fold change >2 or <0.5 and *p*-value (*t* test) of <0.05 comparing different age samples. Color bar represents the relative intensity of identified proteins from −4 to 4. **C** and **D** Heatmap depicting the levels of differentially identified oxidations and AA substitutions among different ages. The graphs show the relative intensity of differentially expressed modifications. Color bar represents the relative intensity of identified proteins from −6 to 6. **E** and **F** The scatter plot graphs showing all differentially expressed oxidations that are potential diagnostic markers for aging. Comparison of oxidation differences among children group, young age, and senior group. The horizontal line in the middle of the data represents the median, the upper and lower color lines represent the data quartile range, and the upper part of the data represents the significance between groups. Data are presented as mean ± SD. As the number of labels increases, the significance increases. (*p <0.05, **p <0.01, ***p <0.005, ****p <0.001). **G** 14 modifications changed trend with age. The expression of 11 oxidation modifications and 3 AA substitutions increased with aging. **H** and** I** The scatter plot histogram graphs showing 11 differentially expressed oxidation modifications and 3 AA substitutions that are potential diagnostic markers for aging. Comparison of oxidation differences among children group, young age, and senior group. The horizontal line in the middle of the data represents the median, the upper and lower color lines represent the data quartile range, and the upper part of the data represents the significance between groups. Data are presented as mean ± SD. As the number of labels increases, the significance increases. (*p <0.05, **p <0.01, ***p <0.005, ****p <0.001). **J** The ROC plot of 4 oxidations and an AA substitution

Among all modifications, we found that oxidations and AA substitutions had obvious trends reflecting the increase in age. Unsupervised cluster analysis was performed on the global modifications (n=1480) and all significant (n=242) modifications, and each sample was grouped into its age group (Fig. 2a and b). And cluster analysis showed that it could be an obvious feature to distinguish three different age groups very well. A total of 242 modifications with significant differences using a two-tailed test (*p*-value < 0.05, fold change >2 or <0.5) and the changes in each modification between different groups could be obtained through calculation and statistics. Here we listed changes in 26 oxidations and 58 AA substitutions (Additional file 1: Table S3). And every modification classification was quoted from the UniMod website (http://www.unimod.org/modifications_list.php).

Due to the significant difference in oxidative modifications, and its close correlation with age, we found out all oxidative modifications in total modifications, including oxidative modifications of proline, tryptophan, tyrosine, cysteine, methionine, and other insignificant and non-differential oxidative modifications (34 types in total). Unsupervised cluster analysis showed that it could be a perfect feature to distinguish these different age groups well, and Fig. 2c showed the details. Meanwhile, all amino acid substitutions (n=383) cluster analyses could distinguish different age groups very well (Fig. 2d). Among the significantly up- or down-regulated modifications in the older individuals, most oxidations showed significant changes compared to both children and young people (Fig. 2e, f). Oxidation of methionine was eliminated because it may be affected during the experiment.

To minimize the false-positive results brought by Open-search, for the significantly expressed oxidation modifications and AA substitution obtained, the facticity of these modifications was further checked by refined search identification. All the differential modifications information was added in the modification type, while the other search parameters remain unchanged. Due to the huge amount of calculation when performing a refined search with various modifications, supercomputing was used here. Among them, the expression of 11 oxidation modifications and 3 AA substitutions increased with age signally (Fig. 2g). Most of the comparisons of seniors with others were significantly differential using a two-tailed test (*p*-value <0.001), indicating that these modifications had higher expression than young and children (Fig. 2h, i). The performance of single confirmed candidate markers screened by the refined search analysis, which could be expressed in each group, was qualified both graphically and statistically with the ROC curve method between the senior group and the other two groups (as a whole group) [46]. Wilcoxon Rank-Sum test was used to establish the statistical significance of a single marker and evaluate the significance of the whole ROC curve. Four oxidations and 1 AA substitution’s had an AUC (area under the curve) >0.99 (Fig. 2j), and model performance parameters and Delong test were listed in Additional file 1: Table S4.

After scrambling the data serial numbers between different groups, they were randomly and independently grouped to form new groups, which were screened for differential modification according to the same standard (*p*-value<0.01, fold change >2 or <0.5) to verify the false-positive rate of 11 oxidations and 3 AA substitutions. All the data from 38 all samples (children and young people as a group, 32 samples, the elderly as a group, 6 samples) were randomly divided into two groups with a total of 2,760,681 (${C}_{38}^{6}$) different combinations. Through the same differential screening conditions, the combination under each condition was statistically analyzed. After detailed calculation and statistics, random combinations of ten oxidative modifications and three AA substitutions were obtained (Additional file 1: Table S5). Through the random grouping test, it was found that the randomness of the 14 modifications was found to be approximately 2%, and the reliability was more than 95%. The difference between these 14 modifications in different age groups was less likely to be generated at random.

### Oxidations and oxidized proteins changing in validation cohort

**Fig. 3:**
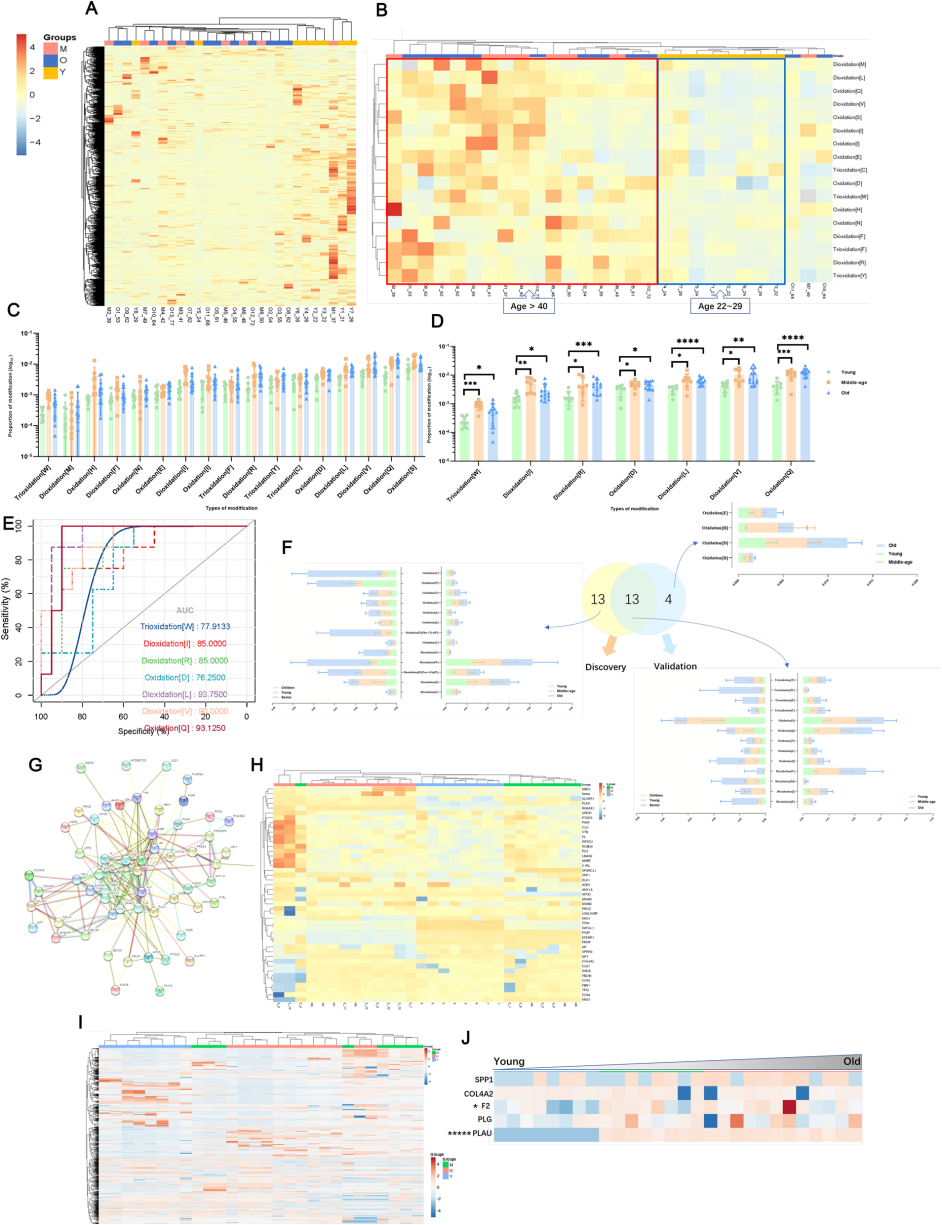
Open and refined search of validation cohorts. **A** Heatmap depicting the levels of global modifications among different ages. The graphs show the relative intensity of young and middle-old group. Color bar represents the relative intensity of identified proteins from −4 to 4. **B** Hierarchical cluster analysis of 17 oxidations which are all belong to post-translational and chemical derivative modifications to the exclusion of artefact modifications. Color bar represents the relative intensity of identified proteins from −4 to 4. **C** The scatter plot histogram graphs showing 17 oxidations fluctuation change. Comparison of oxidation differences among young, middle-age, and old group. **D** The scatter plot histogram graphs showing seven differentially expressed oxidations that are potential diagnostic markers for aging. The horizontal line in the middle of the data represents the median, the upper and lower color lines represent the data quartile range, and the upper part of the data represents the significance between groups. **C** and **D** data are presented as mean ± SD. As the number of labels increases, the significance increases. (*p <0.05, **p <0.01, ***p <0.005, ****p <0.001). **E** The ROC plot of 7oxidation and an AA substitution. **F** The comparison of differentially expressed oxidations in discovery and validation cohorts. Each histogram represents the proportions of each modification, showing how the same modification compares at different ages. **G** STRING protein–protein interaction (PPI) network analysis of oxidative proteins (PPI enrichment p-value< 1.0e-16 ). **H** hierarchical cluster analysis (HCA) of these oxidative proteins distinguishes different ages well. **I** Heatmap of unsupervised cluster analysis depicting the landscape of all identified proteins from old to young. **J** The trends of 5 proteins with aging, PLAU and F2, were differentially expressed using two tailed tests, and the expression increased with aging (*p* value < 0.05, fold change > 2). (*p <0.05, *****p <0.00001)

To verify the oxidative modification in different age groups, we next collected 28 samples from individuals 21–72 years old to repeat the above experimental process. First, using open-search to obtain global modifications, the hierarchical cluster analysis could distinguish young from the middle and the older individuals (Fig. 3a). Given the importance of oxidative modifications, we used 1 oxidation to retrieve by refined search, of which the trend still increased with aging. We found 17 modifications expression increased with aging, belonging to post-translation and chemical derivative modifications to the exclusion of artifacts and hierarchical cluster analysis of 17 oxidations distinguished the young group from the middle-aged and senior people (Fig. 3b, c). There were seven differentially expressed oxidations among young, middle-aged, and old groups that are potential diagnostic markers for aging (Fig. 3d). The performance of single confirmed candidate markers screened by the refined search analysis, which could be expressed in each group, was qualified both graphically and statistically with the ROC curve method between the young group and the other two groups (as a whole group) [46]. Wilcoxon Rank-Sum test was used to establish the statistical significance of a single marker and evaluate the significance of the whole ROC curve. Three oxidations had AUC (area under the curve) >0.9 (Fig. 3e), and model performance parameters and Delong test are listed in Additional file 1: Table S6. In the end, we made a comparison between 26 differential oxidations in discovery and 17 oxidations in validation cohorts of which expression quantities were all increasing with aging (Fig. 3f).

As for seven differentially expressed oxidations, we found the proteins to which they belong. The oxidative sites of osteopontin (gene: SPP1) were the highest among all oxidative proteins. Osteopontin is a multifunctional protein, that has important functions in cardiovascular diseases, cancer, diabetes, and kidney stone diseases and in the process of inflammation, biomineralization, cell viability, and wound healing [47–49]. These oxidative osteopontins maybe have been damaged and lost their function, even indicating osteoporosis. Among oxidative proteins, we found 4 proteins belonging to Platelet Amyloid Precursor Protein Pathway using DAVID Bioinformatics Resources, they are coagulation factor II, thrombin(F2), collagen type IV alpha 2 chains (COL4A2), Urokinase-type plasminogen activator (PLAU), plasminogen (PLG). The 4 proteins’ oxidative sites rank was higher, and in the young group, only 2 of the 4 proteins had few oxidative sites, indicating that with aging beginning these proteins may become damaged, even causing age-related pathologies. Meanwhile secreted phosphoprotein 1(SPP1) and collagen type IV alpha 2 chain (COL4A2) also belong to Regulators of Bone Mineralization. (https://david.ncifcrf.gov).We used oxidative proteins to make protein-protein interaction (PPI, PPI enrichment *p*-value < 1.0e-16) and functional enrichments analysis (Fig. 3g and Additional file 1: Fig. S1). To determine the biological relevance of identified protein networks, we used the g:Profiler toolkit. Functional profiling of each protein set was conducted using the GO, KEGG, and WikiPathways databases. The full set of GO molecular function, GO biological process, KEGG, and WikiPathway terms are provided in Additional file 1: Fig. S2.

MS data were acquired in the DDA scan mode for 28 samples, using MaxQuant and Perseus software processing. After normalizing oxidative proteins intensity, hierarchical cluster analysis (HCA) of these oxidative proteins displays showed common trends in different age stages (Fig. 3h). Next, we compared each stage (young-middle, middle-old. Young-old, young-middle and old) to obtain differentially expressed proteins using two-tailed tests (*p*-value < 0.05, fold change > 2 or <0.5). The overlap of the differentially expressed proteins between each group was shown in Additional file 1: Fig. S3. And we used the common differential proteins between O-Y and MO-Y to make functional annotation through DAVID and g:Profiler. We found that the top biological processes are related to signaling receptor binding, negative regulation of endopeptidase activity, and extracellular matrix structural constituent; the top KEGG pathways were renin-angiotensin system, protein digestion and absorption and pathways in cancer (more detailed and other functional profiles were shown in Additional file 1: Fig. S4). Meanwhile, we found the five differentially expressed proteins of M-Y were involved in the KEGG pathway Ras signaling pathway, and else 5 differentially expressed proteins of O-M were involved in the biological process response to oxidative stress (Additional file 1: Fig.S5). And all these proteins were also involved in some immune- and inflammatory-associated pathways (Additional file 1: Fig. S4, S5). Heatmap of unsupervised cluster analysis depicted the levels of all identified proteins intensity from old to young (Fig. 3i), in which the old samples are classified as one closest cluster. Comparing intensity as aging, the five proteins showed an upwards trend with aging (Fig. 3j), PLAU and F2 differentially expressed using two-tailed tests, and their expression incresed with aging (*p*-value < 0.05, fold change > 2).

### Oxidations changing in male rat cohort

**Fig. 4:**
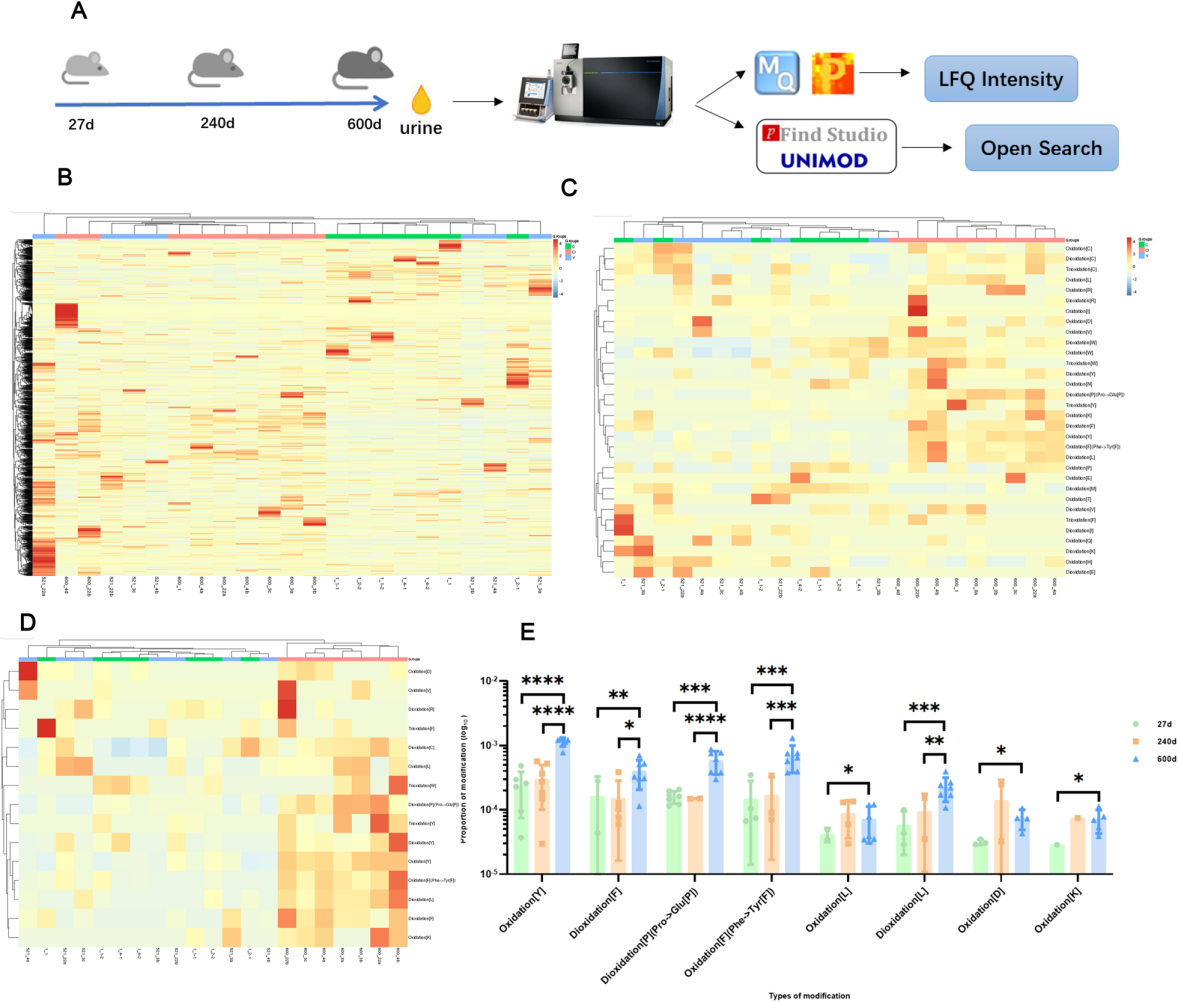
Oxidations changing with aging in male rat cohort. **A** Experimental flow chart of rat cohort. **B** and** C** open-search, unsupervised cluster analyses of both all identified modifications and all oxidations state oxidations in old group is obviously different from the low age groups. **D** 15 oxidations show an upwards trend with aging. **E** 8 oxidations are differential in old from others, and they are almost linear increasing with aging using two tailed tests. Data are presented as mean ± SD. As the number of labels increases, the significance increases. (*p <0.05, **p < 0.01, ***p < 0.005, ****p < 0.001)

Seven male rats of inbreeding were fed from birth, we collected their urine on 27 days, 240 days, and 600 days which is equal to preadolescents, adults, and old as humans [25] (Fig. 4a also shows rat validation flowchart). After open-search, unsupervised cluster analyses of all identified modifications and all oxidations depicted global modifications variation tendency and oxidations changing in old rats significantly distinguished from young and adult stages (Fig. 4b, c). Fifteen non-artificial oxidations showed an upwards trend with aging, hierarchical cluster analysis of these was shown in Fig. 4d. Among them, eight oxidations were differential in old rats compared to other groups, and they showed an almost linear increase with aging as shown in Fig. 4e.

## Discussion

Judging from the large amount of evidence discovered over the years, the damage accumulation theory is one of the most widely accepted where the accumulation of damage is thought to be caused by oxidative stress, and oxidative stress promotes protein modification and senescent cells how to respond to them through protein stabilization mechanisms, including antioxidant enzymes and proteolytic systems [50–53]. Native, functional proteins suffering oxidation and other modifications would be damaged, unfolded, and dysfunctional [7]. Meanwhile, accompanied by dysregulation of UPS, autophagy, and the cross-talk between both systems, these oxidatively modified/damaged by ROS proteins accumulation, age-related pathologies, and the aging process will aggravate [54]. With the normal aging process, damaged proteins accumulate from organs to blood, and urine filtered plasma proteins [9, 10], which obtain damaged proteins. Here, this is the first time to stratify different age people from global modifications, all oxidations can distinguish old people from others; there was a positive correlation between a series of oxidations and age. Unlike previous studies in which a single oxidative site was found on a protein [50, 51, 55–57], this discovery of a series of oxidations reveals oxidative proteins accumulate in old people’s urine. Oxidative proteins and differential protein expression were found to be higher in older group than in other age stages. These proteins are involved in many biological processes including Platelet Amyloid Precursor Protein Pathway, oxidative stress pathway, and some immune and inflammatory associated pathways.

The comprehensive modification of urine protein can provide a new model for monitoring the normal aging of the human body. Because urine is obtained after repeated filtration of blood, its sensitivity can better express the state of the body. Normal urine can also have these oxidized (damaged) proteins, but when their expression or modification changes, they may indicate abnormal aging; and studying the structure of these proteins in urine could provide new insights into the search for drug targets for diseases of aging.

Recently, many studies have demonstrated that a distal body fluids, such as urine, contain organ proteins and even brain-specific proteins, and urine can provide information about the mutation status of disease and uncover its molecular basis [10, 58–60]. However, global modifications in urinary proteome perspective are used to predict the evolving trends in the aging of humans or reveal aging process is the first time. Finally, changes in oxidations and proteins landscape that revealed damaged or oxidative proteins excreted by the body into urine and accumulate in urine as normal aging processing, furthermore, these oxidative proteins can provide information about the prognosis of abnormal aging. The limitation of this study is that the dataset lacks a wider range of ages samples, and we will collect larger datasets to explore why these proteins are modified and whether there are other differential modifications. We purpose that the excretion of these oxidized proteins are excreted through urine to help to maintain homeostasis in the body. These features of old people will guide us in further exploring the aging process, mechanistic studies, and clinical treatments.

## Conclusion

In this study, we found that some modifications were higher in older than younger ages, among which oxidations accounted for a large proportion. These modified proteins may be excreted from the body due to structural changes, resulting in loss of function or even harmful effects on the body. Metabolizing or reducing the accumulation of these proteins may be of great significance for maintaining homeostasis in vivo and even delaying aging and aging-related diseases.

## Supplementary Information

**Additional file 1**. **Table S1.** Detailed information of individuals. **Table S2.** The proportion of modifications occurring at each amino acid. **Table S3. **Differences and statistics of oxidations and AA modifications between different groups. **Table S4. **ROC performance parameters and Delong test in the discovery cohort. **Table S5.** Random grouping calculation results in 14 modifications. **Table S6.** ROC performance parameters and Delong test in validation cohort. **Table S7.** The urine sample processing methods of the published studies in the literature, and the comparison with this method. **Table S8.** The urine sample data collection methods of published studies in the literature and compares them with our method. **Fig. S1.** Oxidative proteins PPI analysis and gene functional analysis using STRING. **Fig. S2.** Functional profiling of each protein set was conducted using the GO, KEGG and WikiPathways databases. **Fig. S3. **The differentially expressed proteins overlap between each group. **Fig. S4.** O-Y-David. BF, MF, CC, and KEGG by David and gene functional analysis by g:Profiler. **Fig. S5.** O-M-David. BF,MF,CC, and KEGG by David and gene functional analysis by g:Profiler. **Fig. S6.** PFind parameters setting (Open-search). **Fig. S7.** a. A illustration of the definition of Species and Sites. As for any modification, “sites” means the quantity of all sites where modifications occur, “species” refers to the type of peptide that is modified. b. Schematic representation of the comparison between the significant modifications via “sites” and “species” aging urinary proteomes. Supplementary site calculate method code: The program codes to extract this information from the “pd.all_result” document.

## Data Availability

All data of children and old groups in the discovery cohort analyzed during this study were downloaded from the ProteomeXchange Consortium via the PRIDE partner repository with the dataset identifiers PXD010469 and PXD004713. Other datasets analyzed during the current study are available in the iProX Datasets under the Project ID: IPX0002313003. (https://www.iprox.org/page/HMV006.html).

## Abbreviations

UPS: Ubiquitin-proteasomal system
DDA: Data dependent acquisition
AA: Amino acid
LC-MS/MS: Liquid chromatography–tandem mass spectrometry
ROC: Receiver operating characteristic curve
AUC: Area Under roc Curve
KEGG: Kyoto Encyclopedia of Genes and Genomes
HCA: Hierarchical Cluster Analysis
LFQ: Label-free quantitation
FDR: False discovery rate
CV: Coefficient of variation
PCA: Principal components analysis
